# A novel PRP-filled collagen tube reliably clinically restores meaningful sensory and motor function across long nerve gaps with long repair delays and in older patients: a case series

**DOI:** 10.21203/rs.3.rs-9334221/v1

**Published:** 2026-04-20

**Authors:** Damien Paul Kuffler, Ivan J. Sosa, Onix Reyes, Chrisatian A. Foy

**Affiliations:** University of Puerto Rico, Medical Sciences Campus; University of Puerto Rico, Medical Sciences Campus; Doctors Center Hospital Maniti; University of Puerto Rico, Medical Sciences Campus

**Keywords:** autograft, nerve regeneration, nerve trauma, platelets, PRP

## Abstract

**Background:**

Autografts, the clinical “gold standard” technique for restoring meaningful function across peripheral nerve gaps, have limitations, including the need to sacrifice a sensory nerve and being reliably effective only for short gaps (< 4 cm), short repair delays (< 5 months), and young patients (< 25). Further, as the value of any of these three variables increases, recovery decreases, and when the values of two or all three increase simultaneously, recovery is minimal or none.

**Method:**

Clinically, upper-extremity nerve gaps were bridged with a novel autologous PRP-filled collagen tube to determine whether this induced meaningful sensory and motor function without the limitations on recovery seen by autografts due to increasing gap length, repair delay, and patient age.

**Results:**

Repairs were performed on nine nerves in eight patients, aged 18–58 years (mean 35.8 years), with gaps 5–12 cm (mean 6.4) long and repair delays of 0.1–3.25 years (mean 0.94). Meaningful motor function was restored to 44% of the nerves, while 67% developed meaningful sensory function, including 100% with sensitivity to 4–8 different types of sensory stimuli, and 78% with fingertip static 2-point discrimination ≤ 15 mm. These recoveries developed despite each patient having the values of 2–3 of the variables that restrict recovery, simultaneously large.

**Conclusions:**

Without sacrificing a sensory nerve, bridging nerve gaps with a novel PRP within a collagen tube, a technique shown to reliably and rapidly eliminate chronic neuropathic pain in each patient, restores meaningful sensory and motor function despite simultaneously long nerve gaps and long repair delays in older patients, conditions where autografts and other techniques exert little to no effect. Further testing will determine the potential limitations of this technique and whether further modifications to the PRP can induce more extensive recovery.

## INTRODUCTION

Although autografts are the clinical “gold standard” technique for repairing nerve gaps and restoring sensory and motor function,[32] their efficacy in reliably restoring meaningful sensory and motor function requires sacrificing a sensory nerve and is limited to short nerve gaps, short repair delays, and young patients.[26, 41, 7, 32, 36] As the value of any of these three variables increases, the extent of recovery decreases, with recovery limited to none when the values of 2–3 of the variables are simultaneously large. [15, 7, 13] These limitations result in < 50% of autograft nerve repairs restoring meaningful sensory or motor function recovery.[32, 15, 48] Therefore, most patients with high values for any of the three variables are not offered nerve repair surgery and suffer permanent loss of function and chronic neuropathic pain.

Novel nerve gap repair techniques are needed that do not require sacrificing a sensory nerve while inducing a larger percentage of patients to recover more meaningful function, especially under conditions where autografts induce minimal to no recovery. This study tested whether, without sacrificing a sensory nerve, bridging nerve gaps with a novel platelet-rich plasma (PRP) within a collagen tube induces meaningful recovery despite simultaneously large gap lengths, repair delays, and patient age.

### Research objectives.

Pilot study to determine whether, without sacrificing a sensory nerve, PRP of a novel composition induces meaningful sensory and motor recovery when the variables of gap length, repair delay, and patient age are singly and simultaneously large.

## MATERIALS AND METHODS

This formal prospective study compared the extent of meaningful sensory and motor recovery after bridging upper-extremity nerve gaps with a novel PRP-filled collagen tube (PRP-repair). This PRP has previously been shown to eliminate chronic neuropathic pain reliably.[25, 24]

### Randomization

Non-randomized, because the study was to determine whether there were any indications of PRP promoting axon regeneration.

### Blinding

No blinding because only one study group.

### Research patients

Patients presenting to the Section of Orthopedic Surgery who required a peripheral nerve gap repair.

### Inclusion criteria.

Patients 18–75 years old with an upper extremity peripheral nerve gap requiring a repair and having at least one of the three variables large, defined as gap length ≥ 4, repair delay ≥ 5 months, or age ≥ 25 years old.

### Surgery

Under full anesthesia and a microscope, the nerve injury sites were exposed, the damaged nerve stumps cut away with a scalpel to where clear nerve fascicles were seen in the proximal nerve stump, and no scarring was seen in the distal stump/s.

### Collagen tubes

Before FDA-approved collagen tubes were commercially available, they were made from 2x8 cm collagen sheets (Veritas, Synovis Life Technologies, St. Paul, MN), FDA-approved for soft tissue repair. The tubes were created by sewing the sheets around the handle of a surgical tool. Tubes created from 8 cm sheets can be used to repair gaps ≤ 7.6–7.8 cm in length, allowing the proximal and distal nerve stumps to be 1–2 mm inside the PRP-filled collagen tube. For longer gaps, two collagen sheets were sewn end-to-end and then into a tube. Subsequently, NeuroMend collagen tubes (Regenity Biosciences, Oakland, NJ) were used. NeuroMend tubes are easier and faster to work with than sheets, and because they have a longitudinal slit, they can be opened, and the autograft slipped inside, after which they self-close with a 25% overlap. Figure #1.

### Preparing and injecting platelet-rich plasma

**Figure 2:**
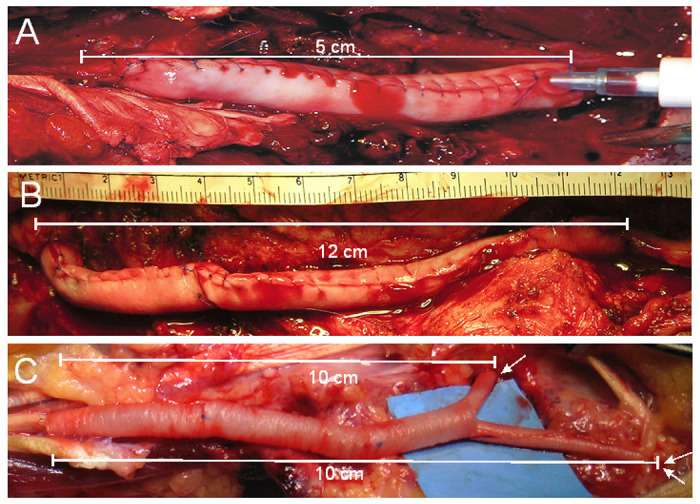
PRP nerve gap repairs. A. Repair of a 5 cm mixed nerve gap with a collagen tube sewn from a sheet of collagen. B. Repair of a 12 cm gap with two collagen sheets sewn end to end. C. Repair of a 10 cm motor nerve gap (tube entry point indicated by a single arrow), and two 10 cm sensory nerve gaps (entry points indicated by two arrows). A Y-ended collagen tube was created by inserting two smaller-diameter NeuroMend tubes into several larger-diameter tubes with overlapping ends.

PRP was prepared and injected as previously described.[25] Briefly, 6 cc of PRP were separated from 55 cc of whole blood using a Gravitation Platelet Separation III (GPS III) centrifuge tube (Zimmer Biomet, Warsaw, IN). After securing a collagen tube between the nerve stumps, PRP plus thrombin was injected to fill the collagen tube. The fibrin polymerized within 20 seconds, and the patient closed. Figure 2 shows two completed autograft/PRP and two PRP repairs.

### Electrodiagnostic studies

Electrodiagnostic studies were performed using surface, needle, and monopolar muscle recording electrodes. Evoked nerve conduction studies (NCS) recorded distal to the injury site evaluated the sensory and motor nerve branches, with the contralateral extremity nerves used to compare distal latencies, nerve conduction velocities, and the evoked response amplitudes.

Electromyographic (EMG) studies of muscles innervated by the repaired nerves sought evidence of denervation potentials, active voluntary muscle contraction, and abnormalities in the motor unit potentials that suggest chronic injury and partial or complete recovery. Axons were considered to have regenerated entirely across the repaired nerve gaps if any surface, needle, or monopolar muscle recordings were positive.

### Motor recovery

Motor recovery was analyzed using voluntary muscle tests and rated according to the modified British Medical Research Council Score, a 6-grade motor function scale (M0-5), with meaningful motor recovery defined as ≥M3.[6, 10] Depending on the location of the repaired nerve injury, each patient underwent 1 to 4 motor function tests. Each muscle group’s motor force was designated as the mean of the forces generated by the different muscle groups innervated by the repaired nerve.

### Sensory recovery

Sensory testing involved the application of eight different types of sensory stimuli: light touch, pinprick, pressure (using Semmes-Weinstein monofilaments), vibration, heat, cold, proprioception, and static 2-point discrimination (s2PD) using a Disk-Criminator (Sensory Management Services LLC, Lutherville, Md.). The extent of sensory recovery was ranked using the Mackinnon-Dellon sensory recovery scale. Meaningful sensory recovery was defined as ≥S3.[6, 10]

### Statistical analysis

Statistical analysis was performed using Excel to determine means and standard deviations.

### Patient follow-up

To ensure consistency, the electrophysiological or physical studies were performed by the same clinicians.

## RESULTS

### Demographics: patients, nerves, and recoveries

The PRP repairs involved 8 patients, all males, and 9 upper-extremity mixed sensory/motor nerves, with 5–12 cm gaps (mean 6.4 ± 1.1), repair delays of 0.1–3.25 years (mean 0.94 ± 1), and aged 18–58 years old (mean 35.8 ± 1. Table 1. The nerves were radial (11%), median (1.1%), and ulnar (67%), with the injuries located at the wrist (11%), forearm (22%), and elbow (67%). The causes of the injuries were sharp objects (22%), mechanical trauma (22%), and gunshot wounds (56%). Of the patients, 22% had 2 simultaneously large variables, 78% had 3 simultaneously large variables, and all had 2 or 3 simultaneously large variables.

### Electrodiagnostic Analysis - evoked potential studies and analysis of reinnervation

Electrical stimulation of the PRP-repaired nerves proximal to the repair site evoked sensory nerve action potentials distal to the repair site recorded with surface or needle electrodes distal to the nerve repair site, with prolonged onset latencies, which decreased over time but had normal conduction velocities and amplitudes. These results indicate that some axons of each repaired nerve regenerated entirely across the repaired gaps.

Although the muscle targets became reinnervated, monopolar electromyogram studies revealed varying degrees of denervation. Thus, action potentials were recorded from reinnervated muscles in each appropriate muscle target, indicated by motor unit action potentials with normal morphology and muscle fiber recruitment patterns, normal insertional electrical activity, and no spontaneous activity at rest.

### Motor recovery

The PRP repairs restored meaningful motor recovery by 44% of the nerves.

### Sensory recovery

Physiological studies showed that PRP repairs led to meaningful sensory recovery by 67% of the nerves, including static fingertip two-point discrimination (s2PD) of ≤ 15 mm developed by 78% of the nerves, and each nerve restoring sensitivity to 4–8 different types of sensory stimuli.

### Complications

No patient suffered any post-operative complications, such as infections.

## DISCUSSION

Autografts, the clinical “gold standard” technique for restoring function across peripheral nerve gaps, suffer significant limitations. Among these are requiring additional surgery to remove an autograft, sacrificing a sensory nerve function, and reliably inducing meaningful recovery only across nerve gaps ≤ 4 cm,[19] repair delays ≤ 5 months,[21, 29] and patients ≤ 25.[21, 29] Further, the extent of recovery decreases rapidly as the value of any of these variables increases, with little to no recovery when 2–3 are simultaneously large. The present study examined the extent of sensory and motor recovery when each patient had the values of at least one of the variables of nerve gap length, repair delay, and age larger than those at which autografts normally induce meaningful sensory and motor recovery.

### Electrodiagnostic studies

Surface electrode stimulation of repaired nerves evoked small compound muscle and sensory action potentials recorded with needle electrodes distal to the repaired nerve from 83.3% of PRP repairs. They had prolonged distal latencies and slow motor nerve conduction velocities, indicating that some axons of each repaired nerve regenerated entirely across the gaps.

Needle EMG of resting reinnervated muscle revealed fibrillations, denervation potentials, and positive sharp waves, indicating abnormal activity of single muscle fibers and absent or reduced voluntary recruitment of motor unit action potentials. Motor unit action potentials had increased phases of the potential, compatible with partial reinnervation. Thus, despite axons reinnervating each denervated muscle, the repaired nerves suffered substantial axonal loss, regenerating entirely across the repaired gaps, resulting in most patients exhibiting incomplete muscle recruitment and motor unit potential morphology compatible with partial reinnervation.

### PRP and axon regeneration

Animal models have shown that PRP increases axon regeneration when injected into autografts,18–27 and allografts,[49] conduits,[9, 1, 34, 17] vein grafts,[28, 22] short axon gaps,[4] onto sheep nerve crush sites,[37] rabbit cornea,[20] surgical nerve injury sites, [27] and leprosy-induced peripheral neuropathy.[3] Clinically, PRP promotes axon regeneration and induces meaningful recovery following an intraneural injection,[12] eliminates palsy with drop foot when applied to the peroneal nerve,[38] improves recovery following carpal tunnel surgery,[31] when applied to the site of a radial nerve primary repair following a four-month repair delay,[12] when injected into the common peroneal nerve,[38] following pudendal nerve decompression,[18] and when injected intraperineurially into nerves with Mycobacterium leprae-induced peripheral neuropathy, which induces sensory axon regeneration and sensory recovery.[3] No clinical study has clinically tested the efficacy of PRP within a conduit bridging long nerve gaps in promoting axon regeneration and restoring function.

### Motor and sensory recovery

A recent systematic literature review found that for short (5–30 mm) and long (50–70 mm) nerve gaps, autografts restored sensory and motor function to 71.8% and 56.0% of nerves, respectively.[26] In the present study, for 5–12 cm gaps with a mean gap length of 6.4 cm, PRP repairs restored meaningful sensory function to 67% of nerves, similar to the number induced by autografts across short nerve gaps.[26] The potent axon-regeneration-promoting effect of PRP is also shown by 78% of the nerves developing static 2-point discrimination ≤ 15 mm and 100% developing sensitivity to 4–8 different types of sensory stimuli. These recoveries were despite all the patients having values of 2–3 variables simultaneously large.

The PRP repairs restored meaningful motor recovery to 44% of the nerves. This lower recovery rate compared to that of sensory nerves is consistent with studies showing that regenerating sensory neurons induce more extensive recovery than motor neurons.[39, 5] Contributing to this reduced recovery is the mean 1-year repair delay, which results in irreversible intrinsic hand muscle atrophy, the muscles’ losing their capacity to generate greater force,[47, 8, 14, 23] and axotomized motor neurons extending fewer and shorter axons.[43, 40] This reduced motor recovery supports the well-established recommendation that achieving maximum functional recovery requires minimizing repair delays.[35, 40]

### Why is this novel PRP more effective in promoting meaningful recovery than PRP in other studies?

Many studies have examined the efficacy of PRP in promoting axon regeneration. The platelet concentration used in rat [4] and rabbit [45, 51] studies is typically 2.5-3.5-fold to 7.5-8.5-fold higher than in whole blood,[2, 30] with further increases, possibly delaying healing.[45, 11] We propose that the greater efficacy of this novel PRP than that used in animal models and other clinical studies is due to how the PRP was prepared and applied. (1) The novel PRP was prepared using the Zimmer Biomet GPS III PRP separation system, which increases the PRP platelet concentration 9.3-fold, several-fold higher than used in most studies.[16] (2) The GPS III device also increases the PRP leukocyte concentration 5-fold, with leukocyte-rich PRP inducing more extensive axon regeneration than leukocyte-poor PRP.[42] (3) Compared to PRP from other devices, this PRP has a higher concentration of bioactive factors. (4) A larger PRP volume was used, up to 6 cc per site vs. <1 cc for most other studies.[42] (5) The PRP was surrounded by a collagen tube, which, in the rat, enhances PRP efficacy.[42]

### Mechanisms of PRP action

We propose that PRP promotes meaningful sensory and motor recovery by platelet-released factors that induce Schwann cell proliferation [50, 46, 33] and the release of neurotrophic factors that increase the number and length of axons neurons extend.[50] Simultaneously, platelet-released VEGF promotes vascularization, which is essential for supporting axon regeneration. In addition, other platelet-released factors act directly on neurons, freeing them from the intrinsic and extrinsic restrictions imposed by long gaps, long repair delays, and increasing age, thereby increasing the capacity of sensory and motor neurons to extend longer axons.[44] Further studies are required to understand which platelet-released factors underlie these influences, their mechanisms of action, whether PRP efficacy is limited by gap length, repair delay, and patient age, and whether the PRP can be improved to increase its efficacy.

## CONCLUSIONS

Electrophysiological and physiological studies show that bridging nerve gaps clinically with a novel PRP-filled collagen tube promotes axon regeneration and functional recovery without sacrificing a sensory nerve. Meaningful motor function was restored by 44%, and meaningful sensory function by 67% of the nerves. Consistent with other studies, the higher level of sensory recovery shows that denervated sensory axons retained a greater capacity to regenerate than motor neurons. However, we also propose that the lower level of motor recovery is due to the mean 1-year repair delays leading to irreversible intrinsic muscle atrophy, the muscles losing their capacity to regenerate full motor force, and the reduced capacity of axotomized motor neurons to extend long axons. These sensory and motor recoveries developed despite all patients having simultaneously large the values of 2–3 of the variables that restrict or prevent recovery. This novel PRP was previously shown to reliably and rapidly eliminate chronic neuropathic pain. Therefore, these results show that platelet-released factors not only rapidly and reliably eliminate chronic neuropathic pain but also induce meaningful sensory and motor recovery by overcoming the intrinsic and extrinsic limitations imposed on neurons, including by increasing gap length, repair delay, and patient age on axon regeneration and recovery. Further testing will determine the potential limitations of this technique, whether modifying the PRP can induce more extensive recovery, and a blinded prospective study will compare the efficacy of this novel PRP vs. autografts in simultaneously restoring function and reducing chronic neuropathic pain, and determine the limits of this PRP in terms of maximum gap length, repair delay, and patient age.

### Study limitations

The sample size was adequate for statistical analysis. However, its small size is a limitation and was not large enough to determine whether the efficacy of the PRP decreased with increasing gap length, repair delay, and patient age. Further, generalization of the electrophysiological results may be limited by the wide range of time intervals between nerve injuries and performance studies. While some might consider the wide range of gap lengths, repair delays, and patient ages a limitation, it was a benefit, as the results show that the technique is effective across a wide range of mixed variables.

### Adverse events.

No patient suffered any adverse event, such as infections.

## Figures and Tables

**Figure 1:**
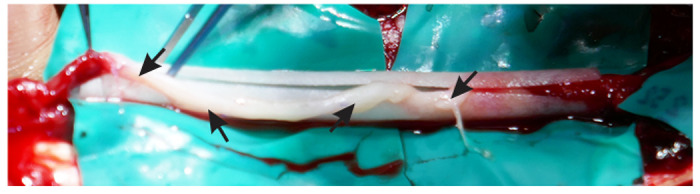
An open 5 cm long NeuroMend collagen tube with a sural nerve autograft (arrows) being inserted into the tube.

**Table 1:** Demographics and recoveries

Demographics / Recoveries	PRP repairs
# patients	8
gender	male
# repaired nerves	9
gap lengths	5-12 cm (mean 6.4 SD 1.1)
repair delay	0.1-3.25 year (mean 0.94 SD 1)
patient ages	18-58 year (mean 35.8 SD 1)
motor recovery ≥M3	44%
sensory recovery ≥S3	67%
# nerve with sensitivity to 4-8 types of sensory stimuli	100%
2-point discrimination ≤ 15 mm	70%
# with values of 2-3 variables simultaneously large	100%
